# Prolonged Amphetamine Treatments Cause Long-Term Decrease of Dopamine Uptake in Cultured Cells

**DOI:** 10.1007/s11064-019-02938-7

**Published:** 2019-12-27

**Authors:** Nafisa Ferdous, Sirisha Kudumala, Serena Sossi, Lucia Carvelli

**Affiliations:** 1 Brain Institute, Florida Atlantic University, Jupiter, FL USA; 2 Harriet L. Wilkes Honors College, Florida Atlantic University, FL Jupiter, USA; 3 Integrated Biology Program, Florida Atlantic University, FL Boca Raton, USA; 4 Department of Biomedical Science, University of North Dakota, Grand Forks, ND USA

## Abstract

Amphetamine (AMPH) is a systemic stimulant used to treat a variety of diseases including Attention Deficit Hyperactive Disorder, narcolepsy and obesity. Previous data showed that by binding to catecholamine transporters, AMPH prevents the reuptake of the neurotransmitters dopamine (DA) and norepinephrine (NE). Because AMPH, either used therapeutically at final concentrations of 1–10 µM or abused as recreational drug (50–200 µM), is taken over long periods of time, we investigated the prolonged effects of this drug on the uptake of DA. We found that, in LLC-PK1 cells stably expressing the human DA transporter (hDAT), pretreatments with 1 or 50 µM AMPH caused significant reduction in DA uptake right after the 15-h pretreatment. Remarkably, after 50 but not 1 µM AMPH pretreatment, we observed a significant reduction in DA uptake also after one, two or three cell divisions. To test whether these long-term effects induced by AMPH where conserved in a model comparable to primordial neuronal cells and native neurons, we used the human neuroblastoma cell line SH-SY5Y cells, which were reported to endogenously express both hDAT and the NE transporter. Pretreatments with 50 µM AMPH caused a significant reduction of DA uptake both right after 15 h and 3 cell divisions followed by neuro-differentiation with retinoic acid (RA) for 5 days. Under these same conditions, AMPH did not change the intracellular concentrations of ATP, ROS and cell viability suggesting, therefore, that the reduction in DA uptake was not cause by AMPH-induced toxicity. Interestingly, while 1 µM AMPH did not cause long-term effects in the LLC-PK1 cells, in the SH-SY5Y cells, it decreased the DA uptake after one, two, but not three, cell divisions and 5-day RA differentiation. These data show that besides the well-known acute effects, AMPH can also produce long-term effects in vitro that are maintained during cell division and transmitted to the daughter cells.

## Introduction

The neurotransmitters dopamine (DA) and norepinephrine (NE) belong to the catecholamine and phenylethylamine families of organic compounds and play an important role in fine-tuning a variety of animal behaviors such as movement, reward, cognition and attention. Following their synthesis, DA and NE are rapidly sequestered inside the neuronal vesicles by the vesicular monoamine transporter (VMAT), where they are packed until a depolarizing stimulus promotes the fusion of vesicles to the cellular membrane and the extracellular release of the neurotransmitters. In the synaptic cleft, DA and NE bind and activate their respective receptors and, thus, propagate dopaminergic and noradrenergic signaling. Although most of the released catecholamines diffuse away from the synapse [1], a good portion binds to the DA and/or NE transporters (DAT and NET) [2, 3]. This step prevents further stimulation of the receptors. Therefore, DAT and NET control the intensity and the duration of the signal propagated by DA and NE. Moreover, when DAT moves DA inside the neurons, it causes cell-membrane depolarization affecting, therefore, neuronal excitability [4, 5].

All substances that induce dependence increase the extracellular concentration of DA and NE [6–8]. Amphetamine (AMPH) for example, performs this task through two different mechanisms. As the chemical structure of AMPH is very similar to that of DA and NE, AMPH is carried inside the neurons by DAT or NET preventing, therefore, the reuptake of these catecholamines [9]. Once inside the neurons, AMPH forces DA and NE out of the storage vesicles by acting on VMAT [10]. The subsequent increase of cytoplasmic DA/NE induces DAT or NET to work in reverse resulting in the efflux of DA/NE into the synaptic cleft [11, 12]. The overall effect is, therefore, the accumulation of larger amounts of extracellular DA/NE with respect to that obtained using DAT or NET inhibitors, such as cocaine or methylphenidate [13].

Previous reports demonstrated that acute and brief (1 min) treatments with AMPH increase the surface expression of DAT [14, 15], whereas brief repeated or longer treatments (5–60 min) cause a decrease of surface expression of DAT, as measured by reduced DA uptake activity and DAT-mediated inward currents [16– 19]. These effects were thought most likely be due to reallocation of the transporter from the plasma membrane to intracellular compartments [16, 20, 21], though German et al. reported that in vivo treatments with AMPH reduced the transport activity of murine striatal DAT without concomitant internalization of the transporter in ex vivo preparations [22].

The data mentioned above are examples of the several studies carried out over the last decades on the effects that acute AMPH treatments generate on DAT or NET activity. On the other hand, there are few data describing the effects generated by prolonged [23] AMPH treatments on the two transporters. Here we investigated the effects caused by 15-h treatments with 1 or 50 µM of AMPH on the uptake activity of hDAT heterologously expressed in the pig kidney cells or in the human neuroblastoma SH-SY5Y cells which endogenously express DAT and NET. We found that after 15 h treatment, both concentrations of AMPH reduced the accumulation of [^3^H]DA inside LLC-PK1 and SH-SY5Y cells. Interestingly, this effect was inherited by the daughter cells up to three cell divisions in the LLC-PK1 cells treated with 50, but not 1 µM AMPH, whereas in the SH-SY5Y cells, both doses caused a significant reduction of [^3^H]DA uptake in daughter cells after three and two cell divisions, respectively. Taken together, these data suggest that AMPH causes long-lasting effects that can be maintained within the cell and inherited during mitosis.

## **Methods**

### Cell Cultures

LLC-PK1 cells stably transfected with hDAT were kindly provided by Dr. Roxanne Vaughan and James Foster (University of North Dakota). LLC-PK1 cells were maintained in α-modified Eagle’s medium (AMEM) containing 2 mM l-glutamine and 200 µg/mL G418 sulfate; whereas SH-SY5Y cells were grown in Dulbecco’s Modified Eagle’s Medium/F12 (DMEM). Both media were supplemented with 5% and 10% fetal bovine serum (FBS), respectively, and 1% penicillin/streptomycin. Cells were passaged according to the following protocol: after removing the media, cells were washed twice with 10 mL sterile phosphate buffer saline (PBS). After the PBS washes, 2 mL of trypsin solution was added to promote cell detachment from the flask. Trypsin was removed by aspiration and the cells were incubated in a 37 °C incubator for 5 mins. Once cells detached, the cell suspension was gently mixed with 10 mL DMEM and collected in a 15 mL tube. Cell pellets were collected by centrifugation at 1500 rpm at 4 °C for 5 min, re-suspended in 1 mL fresh DMEM media and equally divided in T-75 flasks containing 10–15 mL media.

### Uptake Assays

300,000 or 150,000 cells were seeded in 24- and 12-, or 6-well plates, respectively. Different sizes of well-plates were used to accommodate the higher number of cells yielded after two and three cell divisions. Six-seven hours after being plated, cells were treated with 1 μM, 50 μM AMPH or control solution for 15 h. After 15 h, cells were washed three times with PBS and either used to measure [^3^H]DA uptake or grown in fresh media to cross one, two or three cell divisions in order to perform uptake assay in daughter cells. Uptake assays were performed as previously published in Carvelli et al. [5]. Briefly, LLC-PK1 cells were washed three times with Krebs-Ringer HEPES (KRH) buffer. KRH containing 0.1 mM tropolone, 0.1 mM ascorbic acid and 0.1 mM pargyline (KRH + TAP), was added to the wells to inhibit DA degradation and oxidation before performing the uptake assays. Finally, [^3^H]DA (PerkinElmer) was added in each well to obtain a final concentration of 20 nM. After 5 min, cells were washed with cold KRH + TAP three times and lysed with 1% triton solution. Lysates were collected in vials and radioactivity was counted using a β-counter. For SH-SY5Y cells, the experimental paradigm was similar to that utilized for LLC-PK1 cells. Cells plated in 24, 12, or 6 wells plates for 6–7 h were treated with 1 μM, 50 μM AMPH or control solution for 15 h. After the drug was washed off with three washes with PBS, one set of cells was immediately assayed for DA uptake. Another set of cells was let grow to pass one cell division as determined by cell counting. At this point, cells were treated with 10 μM retinoic acid (RA) in low serum media (DMEM containing 1% FBS) for 5 days, during which media was replaced once with fresh media containing 10 μM retinoic acid. Cells that went through two or three cells divisions also received the 5-day RA treatment before being assayed for [^3^H]DA uptake as described above for the LLC-PK1 cells.

### Elisa Experiments

To investigate possible residue of AMPH inside the SH-SY5Y cells, we used the Enzyme-linked Immunosorbent Assay (ELISA) kits (Abnova, TW). These consist of micro-wells coated with polyclonal anti-d-AMPH and d-AMPH conjugated to horseradish peroxidase (HRP). The principle of the assay is based on the competitive binding of AMPH and AMPH-HRP in proportion to their concentration in the reaction mixture. Cells were first detached with trypsin and collected by centrifugation. Then cells were washed with cold PBS three times, re-suspended in PBS to be sonicated (5 pulses for 5 s and then 10 pulses for 10 s) and subjected to centrifugation at 1500×*g* (4000 rpm) for 10 min at 2–8 °C to remove cellular debris. Supernatants were collected and stored at − 20 °C or − 80 °C to avoid loss of bioactivity and contamination. On the day of the experiment, samples were brought to room temperature and 10 μL of each sample, control and AMPH-treated, were incubated with 100 μL dilution of enzyme (Horseradish peroxidase) labeled d-AMPH derivative in micro-plate wells which are coated with fixed amounts of oriented high affinity purified polyclonal antibody. Samples were incubated for 60 min at room temperature in the dark. After removing the enzyme conjugate and washing the wells with 200 µL distilled water, the chromogenic substrate was added followed by an acid stop solution to cease the color produced from the substrate. Finally, the absorbance in each well was read within 1 h at a wavelength of 450 nm.

### ATP and ROS Levels

ATP changes were measured using the CellTiter-Glo Assay kit (Promega) following the protocol recommended in the kit. Briefly, 4000 and 8000 SH-SY5Y cells were plated in white opaque-walled 96-well plates (Falcon Cat. # 353296) in DMEM/F12 media with 10%FBS and 1% PenStrep. After 24 h, cells were treated with 50 µM AMPH or control solution in fresh media for 15 h at 37 °C in a 5% CO_2_ incubator. Cells were washed twice with PBS and phenol-red-free DMEM media was added to the cells. Cells were equilibrated at room temperature for about 30 min before adding the CellTiter-Glo reagents. The luminescent signal from each well, which is proportional to the amount of ATP, was measured using a microplate reader (BioTek Synergy H1) During each experiment, a standard curve was generated using increasing concentrations of ATP (0.025–0.25 µM). Data from each experiment were normalized to control samples with the lower number of cells and analyzed for normality tests and statistical significance using GraphPad Prism 7.04 software.

Reactive Oxygen Species (ROS) were quantified by using the Invitrogen Molecular Probe 2′,7′-dichlorodihydroflorescein diacetate reconstituted in DMSO (H_2_-DCFDA, Cat. # D339). 10,000 or 20,000 SH-SY5Y cells were plated in black 96 well plates (Costar #3603) and treated with 50 µM AMPH or control solution for 15 h. Cells were washed twice with PBS and incubated with 50 µM H_2_-DCFDA for 30 min in phenol-red free and serum free DMEM at 37 °C. The dye was removed, and fresh phenol-red free medium was added to the cells to recover. Cells treated with 50 µM Luperox TBH 70× (Sigma Cat. # 451839) for 1 h following H_2_-DCFDA were used as positive controls. Fluorescent intensity was measured with a microplate reader (BioTek Synergy H1) at excitation and emission wavelength of 480 and 530 nm respectively. Data from each experiment were normalized to control samples with the lower number of cells and analyzed for normality tests and statistical significance using GraphPad Prism 7.04 software.

### Statistics

Data collected form each experiment were imported in Graphpad Prism software and evaluated for normality and statistical significance. Normality tests were performed using KS, Shapiro–Wilk or D’Agostino and Pearson omnibus tests. Statistical significance was performed using the parametric one-way ANOVA test and Bonferroni’s Multiple Comparison post-test. Data were produced by three independent experiments, otherwise indicated, and in each experiment, samples were tested in triplicates or quadruplicates. See figure legends for number of repetitions in each experiment.

## **Results**

### Prolonged Treatment with 50 but not 1 µM Amphetamine Reduces Dopamine Uptake up to 3-Cell Divisions in hDAT Expressing LLC-PK1 Cells

**Fig. 1:**
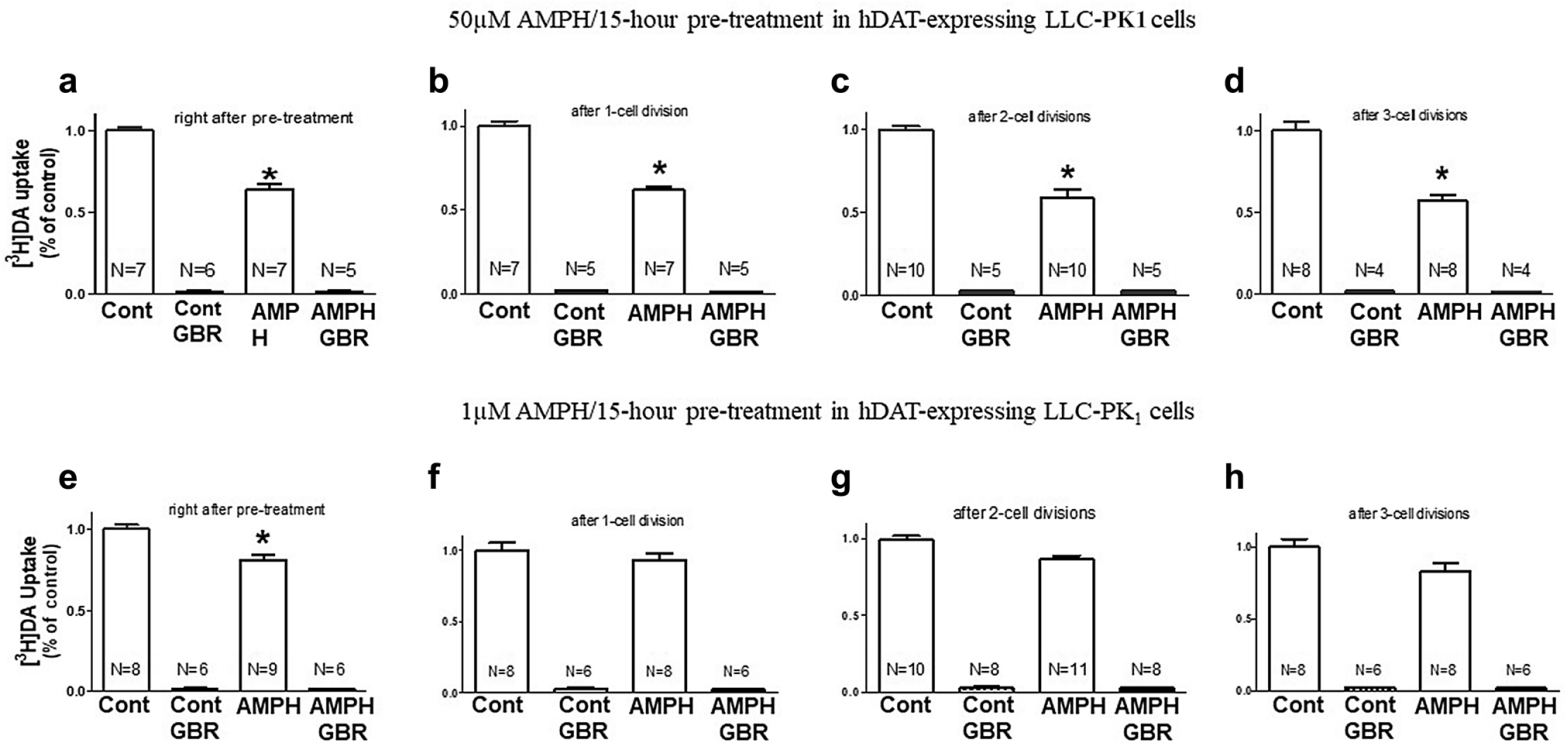
Prolonged treatments with 50 but not 1 µM amphetamine decrease DA uptake up to three cell divisions in LLC-PK1 cells stably expressing hDAT. Samples were pretreated with 50 (**a**–**d**), 1 (**e**–**h**) µM amphetamine (AMPH) or control (Cont) solution for 15 h. After 3 washes, cells were incubated for 5 min with 20 nM [^3^H]DA alone or 20 nM [^3^H]DA and 10 µM GBR 12935. Pretreatments with 50 µM AMPH caused a statistically significant reduction of [^3^H]DA uptake with respect to samples pretreated with control solution both after 15 h and 1–3 cell divisions (**a**–**d**). Pretreatments with 1 µM AMPH caused a statistically significant reduction of [^3^H]DA uptake with respect to samples pretreated with control solution only after 15 h (**e**). GBR 12935 completely blocked [^3^H]DA uptake in both AMPH- or control-pretreated samples (**a**–**h**). Graphs show average values of 3 independent experiments and N represents the total number of wells tested per sample. Data from each graph passed at least one of the 3 normality tests performed (KS, Shapiro–Wilk and D’agostino–Pearson omnibus test) with α = 0.05. Statistic analysis was performed using one-way ANOVA, Bonferroni’s Multiple Comparison test (*p ≤ 0.0001 AMPH vs Cont)

Previous data showed that AMPH pretreatments of 1 or 60 min enhanced or diminished the amount of DAT at the cell membrane, respectively [14, 15, 19]. Here, we investigated whether continuous and prolonged treatments with AMPH caused changes in DA uptake and for how long these changes were maintained. About 260 mg of methamphetamine, taken as single dose by drug abusers, generate a pick of about 7.5 µM in the human blood [24] and concentrations seven times higher, i.e. 52 µM, in rodent brains [25]. Thus, we pretreated hDAT-expressing LLC-PK1 cells with 50 µM AMPH or control solution for 15 h and, then, thoroughly washed the cells to remove the drug. In a subset of samples, [^3^H]DA uptake experiments were immediately performed to quantify the amount of [^3^H]DA accumulated inside the cells. Another subset of cells was grown until their cell count was a value proximal to double, triple or quadruple of the initial number of cells counted before initiating the AMPH pretreatment. This indicates how many cell divisions the cells went through after the AMPH pretreatment. Cells tested right after 15-h AMPH pretreatment exhibited a statistically significant 37 ± 3% reduction in [^3^H]DA uptake with respect to samples pretreated with control solution (Fig. 1a; *p < 0.0001; one-way ANOVA test). Both control- and AMPH-pretreated samples displayed almost no [^3^H]DA uptake when the DAT inhibitor GBR12935 was included during the uptake assay, suggesting therefore that the accumulation of [^3^H]DA inside these cells occurs exclusively through DAT. Interestingly, the other subsets of cells that went through 1, 2 or 3 cell divisions after the AMPH pretreatment also exhibited a statistically significant reduction in [^3^H]DA uptake with respect to control-treated samples, 37 ± 3%, 39 ± 5% and 44 ± 2%, respectively, (Fig. 1b–d; *p < 0.0001; one-way ANOVA test). And, still under these conditions, the [^3^H]DA uptake was blocked by 10 µM GBR12935.

Using previously published data [24, 25], we calculated that concentrations of AMPH for therapeutic use (5–30 mg) yield about 1–6 µM AMPH in the brain. Thus, we investigated whether 1 µM AMPH produced effects similar to those obtained with 50 µM AMPH. As shown in Fig. 1e, 1 µM AMPH caused a statistically significant 20 ± 3% reduction in [^3^H]DA uptake when the assay was performed right after the 15 h pretreatment with AMPH (*p < 0.0001; one-way ANOVA). However, no difference between control- and AMPH-pretreatment was observed after the cells went through 1, 2 or 3 cell divisions (Fig. 1f–h). Taken together these data show that in LLC-PK1, 1 µM AMPH causes short-lived changed in DA uptake, whereas 50 µM AMPH produce a more penetrant effect which is maintained after three cell cycles.

### 1 and 50 µM Amphetamine Reduce Dopamine Uptake up to 3-Cell Divisions in SH-SY5Y

**Fig. 2:**
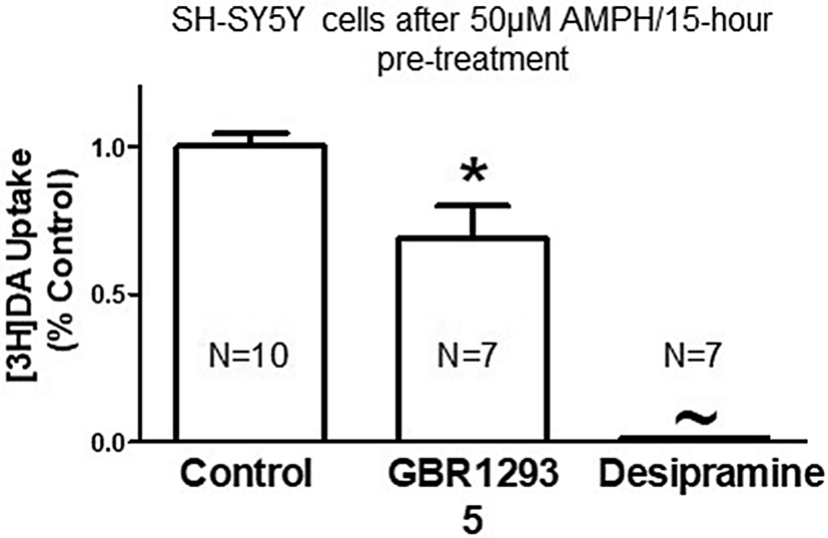
DA uptake in SH-SY5Y cells is efficiently blocked by desipramine rather then GBR12935. SH-SY5Y cells were treated with retinoic acid for 5 days, then washed 3 times and incubated with 20 nM [^3^H]DA alone or 20 nM [^3^H]DA and 100 nM GBR 12935 or 20 nM [^3^H]DA and 100 nM desipramine. Desipramine caused a 98 ± 0.002% reduction in DA uptake whereas, GBR 12935 caused only 31 ± 0.3% reduction. Graph represents average values of 3 independent experiments and N represents the total number of wells tested per sample. Data passed KS (α = 0.05) and Shapiro–Wilk (α = 0.05) normality tests. Statistic analysis was performed using one-way ANOVA, Bonferroni’s Multiple Comparison test (* and ^~^p ≤ 0.005 GBR12935 or desipramine vs Control, respectively)

**Fig. 3:**
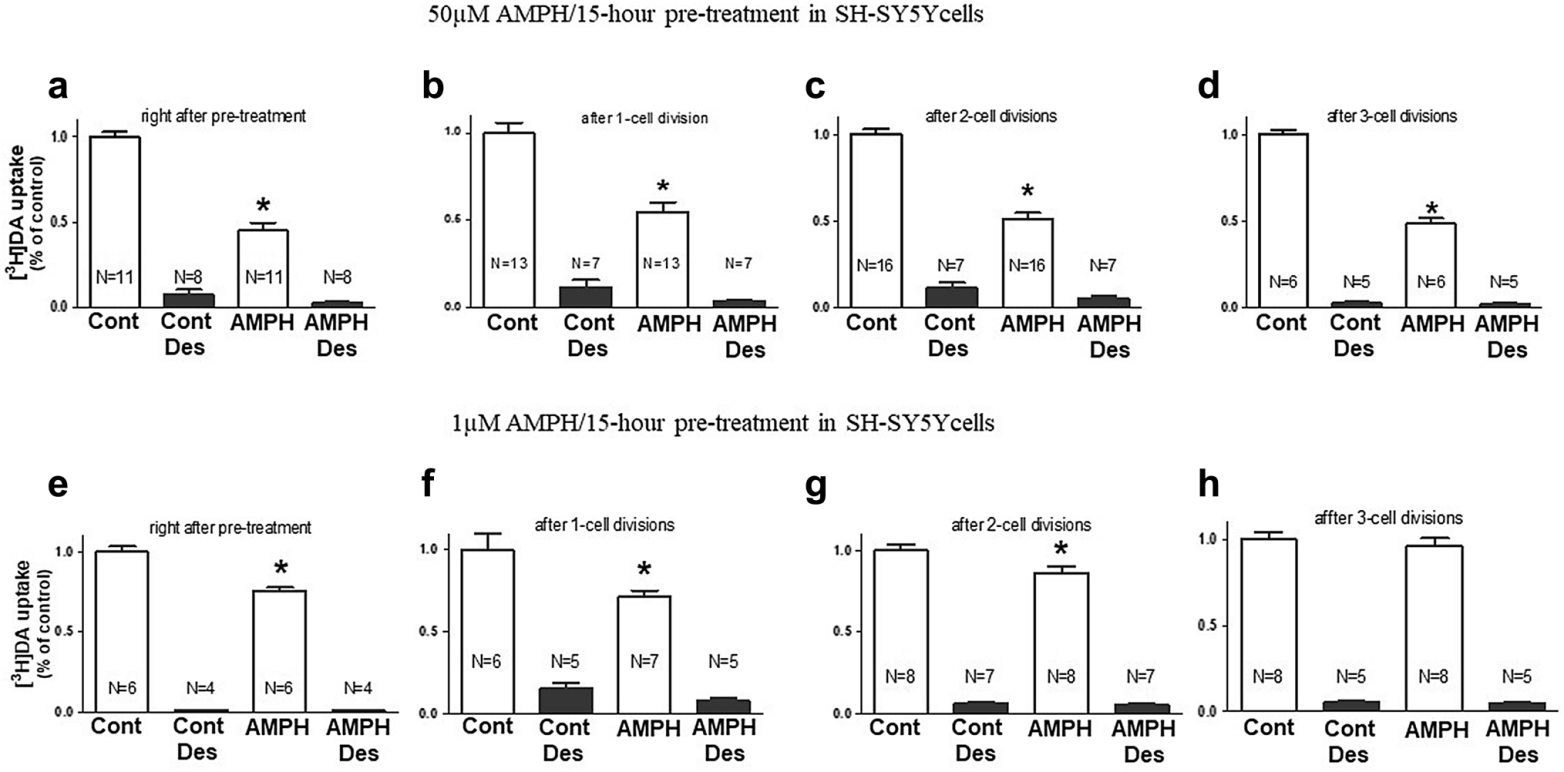
Pretreatment with 50 µM amphetamine (AMPH) caused significant reduction in DA uptake after 1–3 cell divisions whereas, 1 µM AMPH decreased DA uptake after 1–2 but not 3 cell divisions. Samples were pretreated with control (Cont), 50 (**a**–**d**) or 1 (**e**–**h**) µM AMPH for 15 h. After 3 washes, cells were grown to go through 1–3 cell divisions, treated with retinoic acid for 5 days, then thoroughly washed and incubated for 5 min with 20 nM [^3^H]DA alone or 20 nM [^3^H]DA and 100 nM desipramine (Des). Both concentrations of AMPH caused a statistically significant reduction of [^3^H]DA uptake up to 2-cell divisions with respect to samples pretreated with Cont solution (*p ≤ 0.0001; one-way ANOVA, Bonferroni’s Multiple Comparison test). Des completely blocked [^3^H]DA uptake in both AMPH- or Cont-pretreated samples. Data from A, B, C, G and H graphs passed the KS, Shapiro–Wilk and D’agostino–Pearson omnibus normality tests (α = 0.05); **d**–**f** graphs passed the KS normality test (α = 0.05). Graphs represent average values of 3 independent experiments and N is the total number of wells tested per sample

Our initial experiments in LLC-PK1 cells showed that 1 or 50 µM AMPH significantly reduced DA uptake in parent cells and this effect was transmitted in daughter cells only by 50 µM AMPH. To test whether these long-term effects induced by AMPH were conserved in other cell types, we used the human neuroblastoma cell line SH-SY5Y which have been shown to endogenously expresses DAT as well as NET [26–28]. To test if prolonged AMPH pretreatments generate long-term effects on DAT activity also in SH-SY5Y cells, we used the same experimental paradigm used for the LLC-PK1 cells with the addition of one extra step. After the 15 h of AMPH pretreatment, cells were incubated with 10 µM retinoic acid (RA) for 5 days before performing the uptake assay. This step, as previously shown [29, 30], generates a higher number of differentiated neurons. Our pilot data showed that following RA-induced differentiation, 100 nM GBR12935, a specific DAT blocker, only partially blocked the DA uptake whereas, 100 nM desipramine, a specific NET inhibitor, completely blocked DA uptake in the SH-SY5Y cells (Fig. 2). These results suggest that most of the DA in the SH-SY5Y cells is taken up by NET. Therefore, 100 nM desipramine were co-incubated with [^3^H]DA to show the contribution of DAT/NET in our uptake experiments. We found that pretreatment with 50 µM AMPH significantly reduced DA uptake to 55 ± 0.14% (*p < 0.0001; one-way ANOVA) with respect to control-treated cells (Fig. 3a). This effect was maintained after 1- (45 ± 0.2%), 2- (51 ± 0.15%) and 3- (52 ± 0.08%) cell divisions (Fig. 3b–d; *p < 0.0001; one-way ANOVA). Similarly, pretreatments with 1 µM AMPH caused a statistically significant 25 ± 0.05% reduction (*p < 0.0001; one-way ANOVA) in DA uptake after 15-h AMPH pretreatment (Fig. 3e). Remarkably, we found that the effect of 1 µM AMPH was also inherited in daughter cells after 1- (26 ± 0.05%) and 2-cell divisions (15 ± 0.1%), but not after 3-cell divisions (Fig. 3f–h; *p < 0.0001; one-way ANOVA). 100 nM desipramine, co-incubated with 20 nM [^3^H]DA, caused 88–98% inhibition of uptake throughout our experiments (Fig. 3).

Although unlikely, the AMPH-induced reduction of DA uptake observed after the three cell divisions could be caused by residual AMPH not properly washed out from our samples. In this regards, it is worthwhile to note that the DA uptake experiments done after 1, 2 or 3 cell divisions, were performed after 1, 3 or 4 days from the AMPH pretreatment in the LLC-PK1 cells, and after 6, 8 or 9 days in the SH-SY5Y cells. Moreover, before the uptake assay, cells were subjected to changes of media and thoroughly washed with KRH buffer. Nevertheless, we tested the possibility that residual AMPH concentrations were left in our samples by measuring the amount of AMPH present in the SH-SY5Y cells after 15 h of 50 µM AMPH pretreatment and washed three times as we did during the uptake experiments. Using Elisa kits, we found that the concentration of AMPH in cells pretreated with AMPH was 0.013 ± 0.001 pM and this value was comparable to the amount of AMPH, 0.01 ± 0.003 pM, measured in cells pretreated with control solution which never encountered AMPH. These results show that the AMPH-induced reduction of DA uptake measured in our assays (Figs. 1 and 3) is not caused by residual AMPH left in the cell cultures as proved by our Elisa data.

Taken together, these data suggest that prolonged AMPH exposure in cultured cells expressing hDAT and/or hNET exogenously or endogenously causes long-term effects in the uptake activity of the transporters that are maintained during cell divisions.

### Prolonged Treatments with 50 µM Amphetamine do not Affect Cell Viability

Previous data showed that 24-h treatment with 3 mM AMPH cause cell toxicity and consequent death in the SH-SY5Y cells [31]. In our study, we used much lower concentrations of AMPH (1 or 50 µM) for 15 h. Nonetheless, we reasoned to test whether, under our experimental conditions, AMPH caused cell death. If that were the case, then the reduction of DA uptake observed after 15-h treatment and up to 3-cell divisions would be the result of a reduced number of DAT/NET-expressing cells. Thus, during our uptake experiments, extra samples were used in parallel to assess cell viability and to count the number of SH-SY5Y cells that went through the 15-h pretreatment with 50 µM AMPH or control solution. Cell viability, assessed by trypan blue staining combined with an automated cell counter, was in the range of 99–100% for both control- and AMPH-treated samples (Table 1). As shown in the same table, no difference in cell counting between the two groups was observed neither after 15-h or 1, 2, or 3-cell divisions. Moreover, imaging data showed that AMPH did not induce any obvious change in cell morphology in both SH-SY5Y and LLC-PK1 cells (data not shown). These results suggest that 50 µM AMPH for 15 h do not cause cell death in both LLC-PK1 and SH-SY5Y cells.

**Table 1:** Prolonged treatment with 50 µM amphetamine do not affect cell viability

	After 15-h treatment		After 1-cell division		After 2-cell division		After 3-cell division	
	Cell count	Viability (%)	Cell count	Viability (%)	Cell count	Viability (%)	Cell count	Viability (%)
Control	197,000 ± 6000	100	417,000 ± 18,000	100	720,000 ± 20,000	99	1,140,000 ± 160,000	99
50 µm AMPH	192,000 ± 7000	100	430,000 ± 10,000	99	707,000 ± 30,000	100	1,045,000 ± 55,000	99

SH-SY5Y cells were pretreated with control or amphetamine (AMPH) for 15 h. Some samples were differenciated for 5 days with retinoic acid (RA) and then used for cell counting and viability tests (after 15-h treatment), whereas others were grown to go 1–3 cell divisions, treated with RA to induce neuronal differentiation and then tested for viability and cell counting. Data are representative of 3 independent experiments and in each experiment, samples were tested in duplicates

### Prolonged Treatments with 50 µM Amphetamine do not Change Intracellular ATP or ROS in SH-SY5Y Cells

**Fig. 4:**
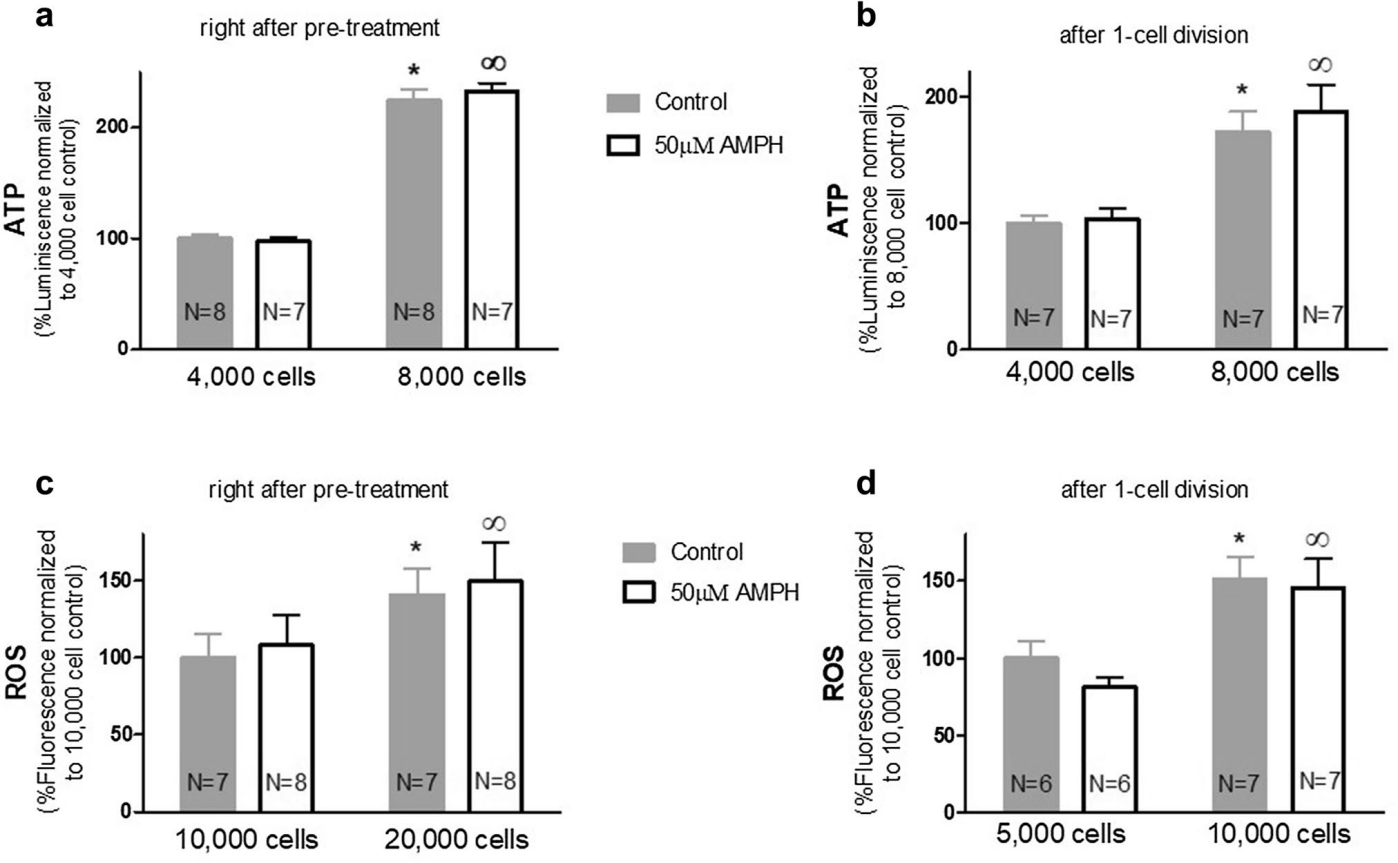
Prolonged treatments with 50 µM amphetamine do not alter intracellular concentrations of ATP or ROS. SH-SY5Y cells were pretreated with control or amphetamine (AMPH). After 15 h, cells were washed 3 times and the intracellular concentrations of ATP (**a**) or ROS (**c**) were measured. In another subgroup of samples, ATP or ROS were measured after the cells went through 1-cell division (**b**–**d**). Graphs represent average values of 2 independent experiments and N is the total number of repetitions per sample. The number of cells in the X axis represent the amount of cells seeded in each well. Data in graphs B and D passed KS, Shapiro–Wilk and D’agostino–Pearson omnibus tests with α = 0.05; whereas, data in A and C graphs passed KS and Shapiro–Wilk tests with α = 0.05. The one-way ANOVA-Bonferroni’s Multiple Comparison test showed significant differences among the number of cells seeded but not between treatments. *p ≤ 0.0001 for Cont-8000 vs Cont-4000 (**a**, **b**) and Cont-20,000/10,000 vs Cont-10,000/5000 cells (**c**, **d**). ^∞^p ≤ 0.0001 for AMPH-8000 vs AMPH-4000 (**a**, **b**) and AMPH-20,000/10,000 vs AMPH-10,000/5000 cells (**c**, **d**)

Administrations of high-dose (10–15 mg/kg) of AMPH and AMPH analogues in rodents increase reactive oxygen species (ROS) and decrease ATP levels [32]. Therefore, we tested whether the highest concentration of AMPH we used, 50 µM, altered ATP and/or ROS production. Using the CellTiter-Glo Assay kit (Promega) we measured the ATP levels in SH-SY5Y cells right after 15 h pretreatment with 50 µM AMPH or after 1-cell division, and found no change with respect to control-treated cells (Fig. 4a, b, compare white bars with gray bars). On the other hand, the ATP values doubled in samples containing twice as much the number of cells (*p < 0.001 and ^∞^p < 0.001; one-way ANOVA, Bonferroni post-test). These experiments show that while the luminescence signal, which is representative of intracellular ATP, is proportional to the number of cells tested, the treatment with AMPH does not change the intracellular levels of ATP.

Next, we tested whether prolonged treatments with 50 µM AMPH changed the intracellular amount of ROS by using the 2′,7′-dichlorodihydroflorescein diacetate (H_2_-DCFDA) molecular probe (Invitrogen). H_2_-DCFDA is cell permeant and, once inside the cell, its acetate groups are removed by intracellular esterases. If ROS are available, oxidation occurs and the fluorescent 2′,7′-dichlorofluorescin (DCF) is produced. Thus, an increase in fluorescence reflects higher amounts of intracellular ROS. As shown in Fig. 4c and d, no significant difference was observed between control- and AMPH-treated SH-SY5Y cells neither after 15 h treatment nor 1-cell division (compare white bars with gray bars). However, also in this case, a significant increase of ROS levels was detected in samples containing a higher number of cells (*p < 0.001 and ^∞^p < 0.001; one-way ANOVA, Bonferroni post-test) confirming the efficacy of the probe. Taken together, these data show that, when exposed to 50 µM AMPH for 15 h, the SH-SY5Y cells do not exhibit significant changes in ROS or ATP.

## Discussion

Since it was discovered, AMPH has been used to treat a variety of mental and physical conditions. Currently, low concentrations of AMPH (5–30 mg/pill) are successfully used to treat patients affected with narcolepsy, chronic fatigue syndrome and those affected with attention deficit disorders. On the other hand, data suggesting systemic toxicity caused by elevated and prolonged use of AMPH (260–1000 mg) have been reported both in animal models and postmortem human samples [33]. These opposite outcomes suggest that the concentration of AMPH is a crucial factor in determining the final effects of AMPH.

It is well established that AMPH increases neurotransmission of DA and NE by competing with the transport of these catecholamines and by displacing their storage vesicles. As a matter of fact, DAT and NET are direct targets of AMPH [34]. Moreover, previous reports have demonstrated that AMPH also alters the amount of DA removed from the synaptic cleft by changing the number of DAT at the plasma membrane [35]. For example, in rat striatal synaptosomes 5 µM AMPH increased surface DAT within 30 s and up 1-min following drug exposure [14]. On the other hand, 1-h treatment with 2 µM AMPH caused loss of DAT on the cell membrane which resulted in 40% reduction of DA uptake [16]. Under these conditions, the reduction of DA uptake caused by AMPH was DAT mediated and not produced by passive membrane diffusion of AMPH, since co-incubation with cocaine, a specific DAT blocker, prevented the AMPH-induced effects on DA uptake. All these data demonstrate that the alterations caused by short treatments of AMPH on DAT function have been well established. However, no study has been performed to test the effects that prolonged AMPH treatments have on the catecholamine transporters. Here we investigated the effect of 15-h continuous exposure of AMPH in cells expressing DAT and/or NET. We used 1 and 50 µM of AMPH because previous data showed that a single dose between 260 and 1000 mg methamphetamine (METH), which are the range of doses used by METH abusers, produce a pick of 7.5–28.8 µM METH in human blood [24]. And since the brain:serum ratio for AMPH measured in rats is about 7:1 [25], we calculated that the amount of AMPH in the brain of AMPH abuser is about 50–200 µM. Similarly, people who use 5–30 mg AMPH as therapeutic treatment are predicted to get about 1–6 µM AMPH in their brain.

To ensure that our results were representative of AMPH acting specifically at the catecholamine transporters, we first investigated the effects that 15-h pretreatments with 1 or 50 µM AMPH produced in LLC-PK1 cells stably expressing hDAT. At the end of the treatment and after thoroughly washing out AMPH from the samples, we found that both concentrations caused a significant and comparable decrease in DAT activity as measured by quantifying the radioactive [^3^H]DA accumulated inside the cells. Previous studies reported that acute or short-lasting treatments with AMPH reduces DAT activity by reallocating the transporter from the plasma membrane into intracellular compartments [16, 20, 21, 35]. We can speculate that also 15-h pretreatments with AMPH lower the capability of cells to reuptake DA by increasing DAT internalization from the cell membrane. Interestingly though, we observed that the reduced DA uptake observed in LLC-PK1 cells pretreated with 50 but not 1 µM AMPH was maintained up to three cell divisions, suggesting therefore, that continuous exposure to 50 µM AMPH also produce a non-previously reported effect which is retained during mitosis.

The LLC-PK1 cells used in our study were engineered to stably express hDAT. Thus, although integrated into the cell genome, the hDAT gene is not normally expressed in these cells. For this reason, we investigated whether the long-term effects generated by AMPH were also observed in a more physiologically relevant cell line. We chose to use the SH-SY5Y cells which are derived from human neuroblastoma cells. These cells contain both epithelial and primordial neuronal cells. The neuronal-type cells are in an early neuronal differentiation stage, characterized by the low presence of markers specific for cholinergic [36, 37], adrenergic and dopaminergic neurons including tyrosine hydroxylase [38], VMAT and DAT [26, 27, 39]. However, after treatment with RA, the SH-SY5Y cells exhibit morphological and biochemical parameters similar to those observed in differentiated neurons [29, 40, 41]. Thus, the SH-SY5Y cells are a good model to study if and how drugs affect neuronal differentiation.

Previous studies showed that 4–11 days of RA treatment increase the number of catecholaminergic neurons in the SH-SY5Y cells [42]. Accordingly, we found that after a 5-day RA treatment, SH-SY5Y cells efficiently accumulated [^3^H]DA and, while the NET specific inhibitor desipramine [43] completely blocked DA uptake, the GBR12935 blocker, which has a higher affinity to DAT than NET [44], only partially diminished DA uptake. This result may have two implications: (I) the RA-treated SH-SY5Y cells express only NET and the reduced ability of 100 nM GBR12935 to block DA uptake (Fig. 2) is due to the reduced affinity this blocker has for NET with respect to DAT; or (II) the RA-treated SH-SY5Y cells express more adrenergic neurons (NET-positive cells) than dopaminergic neurons (DAT-positive cells). In this second scenario, the 30% inhibition of DA uptake observed in presence of 100 nM GBR12935 (Fig. 2) is representative of the reduced number of DAT-expressing neurons in our cultures. Nevertheless, our data show that 100 nM desipramine effectively block DA uptake in the SH-SY5Y cells.

The most important result of our study is that both 1 and 50 µM AMPH pretreatments reduced the transport-mediated DA uptake in SH-SY5Y daughter cells, and this effect occurred several days after the AMPH pretreatment. In fact, after the AMPH pretreatment and before the uptake assay, cells went through 1 to 3 cell divisions followed by a 5-day incubation with RA. This last step was required to increase the number of differentiated catecholaminergic neurons, i.e*.* to increase the number of neurons expressing DAT or NET. These results suggest that prolonged exposure to AMPH during development, when neural progenitor cells are formed, may change the asset of catecholaminergic neurons by reducing the number of neurons expressing DAT/NET or, alternatively, decreasing the expression of catecholamine transporters. One implication of these data is that prolonged use of AMPH during pregnancy might affect neuronal asset in the fetus. However, further experiments are needed to validate these conclusions.

Although unlikely, our experiments left open the possibility that residual concentrations of AMPH, after the 15-h pretreatment, were left behind in the cells. For this reason, we used an Elisa kit to measure the amount of AMPH and found no difference between control- and AMPH-pretreated cells. Therefore, we concluded that the AMPH-induced long-term effects seen in our experiments occurred during the 15-h exposure to the drug but, somehow, was maintained after the removal of AMPH.

We also investigated whether the reduction of DA uptake seen in daughter cells could be the result of cell death or changes in ROS and ATP as previous reports showed that high concentrations of AMPH for 24 h reduce viability of cultured cells [31]. The concentrations of AMPH used in our study, 1 and 50 µM, are much lower than those reported to be lethal in cell cultures (100 µM); yet we investigated whether 50 µM AMPH could induce cell death under our experimental conditions. We found that cells treated with AMPH for 15 h had 99–100% viability, presented no morphological modification and showed no change in cell counting yielded right after the pretreatment or after the various cell divisions with respect to control-treated cells. Moreover, the intracellular levels of ATP or ROS in SH-SY5Y were not changed by 15-h treatment with 50 µM AMPH (Fig. 4). Thus, our data demonstrate that the reduction of DA uptake we measured in our experiments is not due to residual concentrations of AMPH in the cells nor to AMPH-induced cell toxicity, rather they suggest that prolonged treatments with 1–50 µM AMPH may activate unknown mechanisms that can be inherited during cell divisions.

In conclusion, our results suggest that prolonged AMPH exposure during neuronal differentiation may affect the function/expression of DAT/NET in mature neurons. In fact, the experiments performed with SH-SY5Y cells were designed in a way that the treatment with AMPH preceded neuronal differentiation, i.e. when the SH-SY5Y cells exhibit features typical of an early neuronal differentiation stage or neural progenitor cells [36–39], but DAT/NET function was measured after RA-induced differentiation, i.e. when SH-SY5Y cells exhibit both morphological and biochemical features similar to those observed in differentiated neurons [29, 40–42]. Thus, we speculate that prolonged AMPH exposures during proliferation and differentiation of primordial neuronal cells change DA reuptake in mature neurons.
